# Altered ECM deposition and cell adhesion signaling in a human cortical organoid model of fragile X syndrome

**DOI:** 10.1186/s13041-026-01280-8

**Published:** 2026-04-15

**Authors:** Sapir Havusha-Laufer, Venkat Raghavan Krishnaswamy, Noy Krugliak-Shechter, Anjana Shenoy, Maayan Karlinski Zur, Liron Kuznitsov-Yanovsky, Inna Solomonov, Jacob H. Hanna, Irit Sagi, Dalit Ben Yosef

**Affiliations:** 1 Department of Immunology and Regenerative Biology, Weizmann Institute of Science, 7610001 Rehovot, Israel; 2 Institution of Reproduction and IVF & CORAL- Center of Regeneration and Longevity Tel-Aviv Sourasky Medical Center, Tel-Aviv, Israel; 3 Department of Cell and Developmental Biology Sackler Faculty of Medicine, Sagol School of Neuroscience, Tel-Aviv University, Tel-Aviv, Israel; 4 Department of Molecular Genetics, Weizmann Institute of Science, 7610001 Rehovot, Israel

## Abstract

Fragile X Syndrome (FXS) is the most common inherited intellectual disability, and the most common monogenic cause of autism spectrum disorder (ASD). It is caused by epigenetic silencing of the *FMR1* gene leading to the loss of FMRP, an RNA-binding protein that regulates local mRNA translation in neuronal dendrites, crucial for synapse development. Three-dimensional (3D) brain organoid models derived through in vitro differentiation of pluripotent stem cells offer a powerful tool to dissect the underlying mechanisms of neurodevelopmental disorders. Here, we generated human FXS and control organoids using isogenic human embryonic stem cell clones with and without the FXS mutation. Our results show that mature FXS cortical brain organoids can be derived by inhibiting the TGFβ and Wnt pathways. Moreover, expression analyses including immunofluorescence, qRT-PCR, proteomics and western blotting reveal altered levels of neuronal markers and ECM deposition along with modulated downstream signaling molecules. Interestingly, in silico analysis of proteomics revealed several altered pathways, such as cell adhesion, regulation of neurogenesis and cell cycle that are implicated in FXS. Collectively, our unique FXS-organoids derived from isogenic hESC lines may serve as a model for studying the pathology of FXS disorder and for developing therapeutical intervention.

**Supplementary Information:**

The online version contains supplementary material available at 10.1186/s13041-026-01280-8.

## Introduction

Fragile X Syndrome (FXS) is the most common form of inherited intellectual disability and the leading monogenic cause of autism spectrum disorders (ASD) [1, 2]. It is a neurodevelopmental disorder that may lead to abnormal neural plasticity, autism, and epilepsy. This X-linked disease is caused by a CGG-repeat expansion in the 5’-untranslated region of the Fragile X Messenger Ribonucleoprotein 1 gene (*FMR1*). In FXS fetuses, FMRP is expressed up to 12 weeks of pregnancy [3], followed by its progressive inactivation. As a master gene regulator, FMRP is essential in the critical stages of brain development [4, 5].

As an RNA-binding protein, FMRP regulates mRNA transport, stability, and translation, especially in neural cells [6]. FMRP is present in the dendritic spines and synaptic terminals and is involved in synaptic activity by repressing protein translation [7, 8]. Thus, the absence of FMRP in FXS results in altered patterns of protein synthesis [7, 9, 10] and immature dendritic spines [11–13], leading to impaired signaling in intracellular pathways involved in neural differentiation and maturation [14, 15]. The altered protein translation in FXS eventually leads to impaired functional connectivity which may explain the developmental origin of intellectual disability associated with the condition [1, 2, 16]. Most studies have focused on how the absence of FMRP affects cellular proteins, yet its impact on the secretion, deposition, and functional properties of the extracellular matrix (ECM) remains largely unexplored.

The ECM plays a multifaceted role, encompassing not only its well-established function of providing structural support to cells, but also serving as a reservoir for bioactive molecules, which are believed to exert significant regulatory influence on cell fate and tissue integrity [17]. A growing body of evidence suggests altered ECM deposition in various brain pathologies, including enhanced degradation or excessive deposition [18, 19]. Abnormal ECM remodeling has been observed in neurodevelopmental disorders such as autism spectrum disorders, epilepsy and schizophrenia [20–22]. Given that dysregulation of ECM proteins may contribute to the pathogenesis of FXS, a detailed understanding of these impaired pathways in human models may offer new therapeutic avenues.

Remarkably, the study of the ECM’s contribution to FXS is still an emerging field with limited research available [23–27]. What studies exist have shown altered deposition of ECM proteins, leading to increased excitatory synapse formation and dendritic spine length, which in turn affect synaptic plasticity and memory formation [7, 12, 28]. Importantly, all of these studies have been conducted in animal models, highlighting the need for a relevant, human-based platform.

Human models for FXS studies include 2D models of adult brain neurons [29–31] or neural precursor cells (NPCs) extracted from aborted fetuses [32]. These models are often limited by access and maintenance, and none of them express *FMR1*. Human pluripotent stem cells (hPSCs), including human embryonic stem cells (hESC) or induced pluripotent stem cells (iPSCs) derived from FXS individuals have been shown to be acceptable human models for FXS [31, 33].

We have previously derived two isogenic hESC clones: one that carries the full *FMR1* mutation, and the other that is free of the mutation, i.e. an isogenic control (IC). These clones were derived from the same pre-implantation embryo, without any genetic intervention (see Methods section for more details). These lines generate functional neuronal networks following in-vitro neural differentiation, which demonstrated aberrant molecular and functional mechanisms [34–38]. However, this 2D model provides limited information, in part due to lack of ECM, which is known to be a critical factor in supporting cells and guiding their fate. Therefore, the aim of the current study was to develop and characterize 3D brain organoids derived from our FX and IC hESC lines. Our results show the inherent defects in FXS neurodevelopment and previously unknown contributions of ECM to the pathophysiology of the disease.

## Results

### Enhancing fragile X- brain organoids formation using a guided protocol

**Fig. 1:**
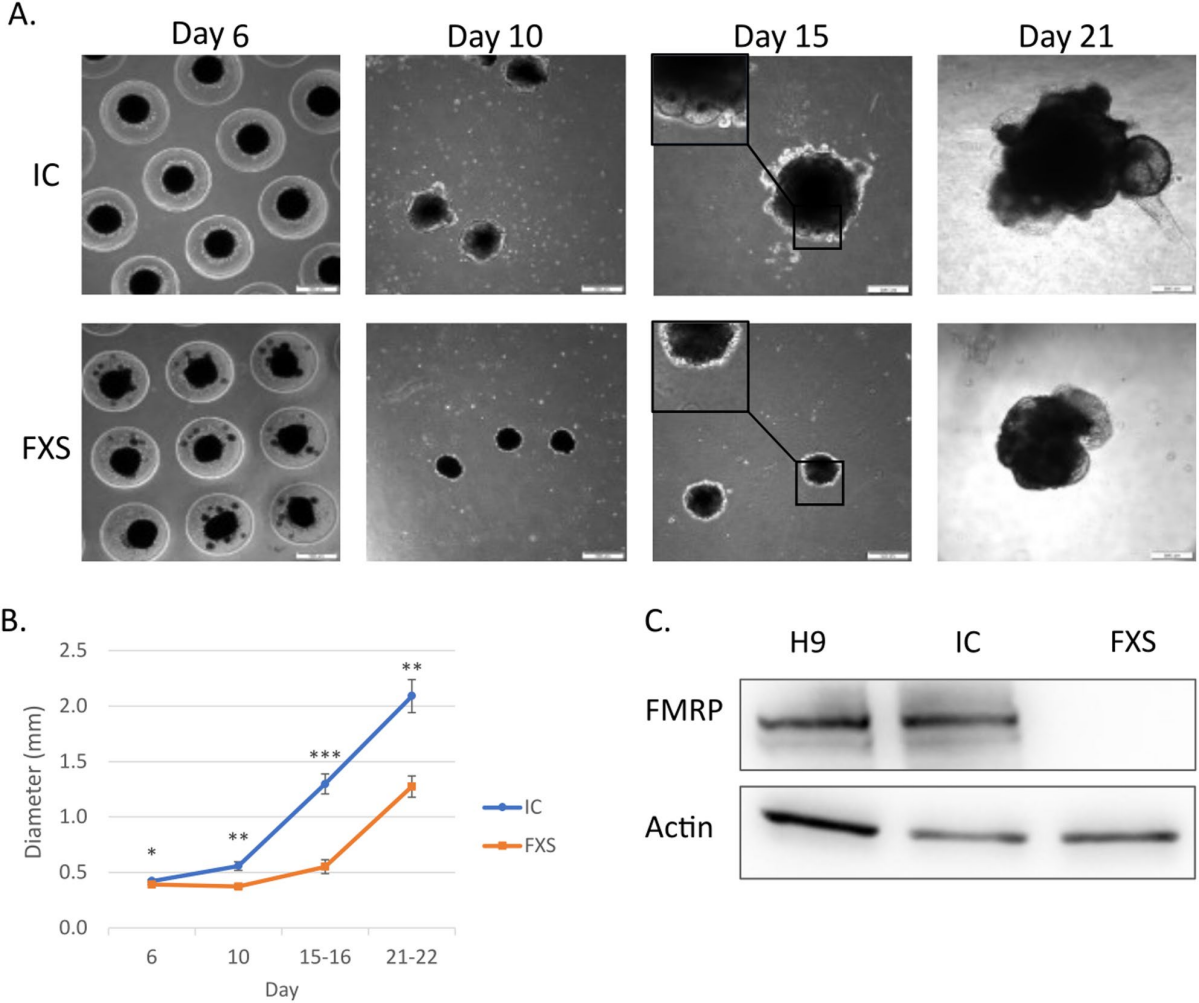
FXS derived cortical organoids display reduced size and complexity.**A** Representative bright- field images of developing brain organoids derived from FXS and IC hESCs clones. 0.75x magnification, Scale bar: 500 μm. **B** Diameter measurements of developing brain organoids. Four independent biological experiments were performed for each cell line, including 27 organoids or more from a minimum of 13 visual fields at each time point. Values are presented as mean ± standard error. *P < 0.05, **P < 0.01, ***P < 0.001. **C** Western blot of FMRP expression in H9 (control wild type hESC line) organoids at day 12, IC and FXS-organoids at day 22. β- actin served as a loading control

To determine how loss of FMRP dysregulates human brain development, we derived 3D brain organoids from the FXS-hESC clone and its IC while implementing the unguided, pan-cerebral protocol published by Lancaster et al. [39, 40] with slight modifications (see Methods for details). Our results indicate that unlike the well-organized organoids developed from IC-hESCs, FXS-hESCs generated significantly smaller cell aggregates with reduced peripheral brightening and diminished or absent neuroepithelial structures (neural rosettes) over three weeks of development (Fig. 1A, B). Moreover, FXS-organoids showed minimal development beyond day 21, and displayed silencing of FMRP by day 22 (Fig. 1C). These results highlight the essential role of FMRP in the organization of brain organoids in vitro.

**Fig. 2:**
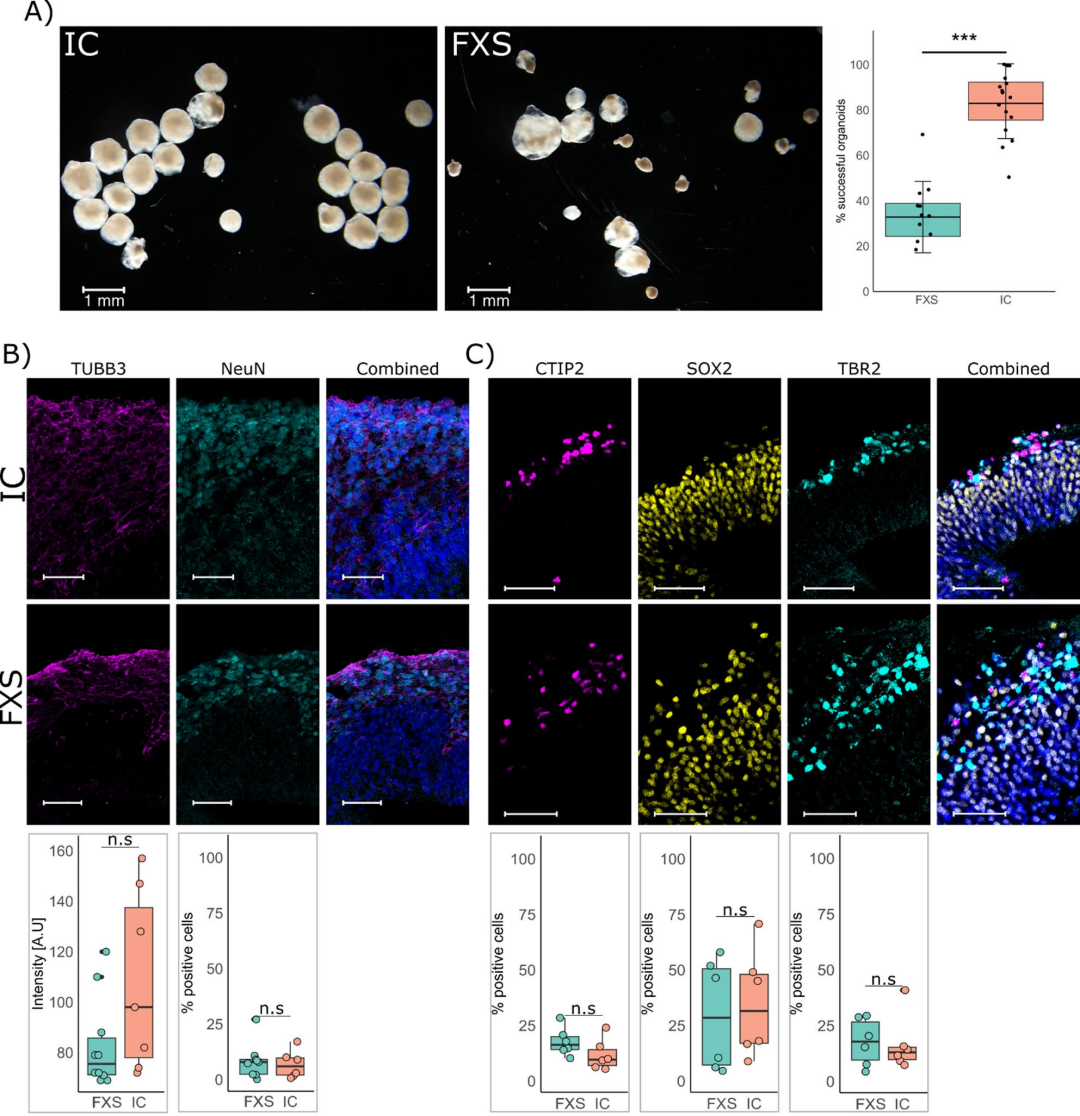
Application of TGFβ and Wnt signaling pathways inhibitors enhance development of FXS-organoids.**A** Stereoscopic imaging of cortical organoids at day 30 and Quantification of successful organoid generation. At least 100 organoids per group were counted across three independent experiments using a stereo microscope. The values represent mean +/- Std and* p* value = 0.003. **B** Representative images and quantification of the neuronal markers NeuN and TUBB3 at day 30 of cortical brain organoids differentiation. Cells were counterstained with DAPI (blue); Scale bar: 40 μm. Three independent biological experiments were performed (n_fxs_=10, n_IC_=7). **C** Representative immunofluorescence staining and quantification of FXS and its IC, showing the expression and localization of early neurons (CTIP2), neural progenitors (SOX2), and intermediate progenitors (TBR2), at day 50 of cortical organoids differentiation. Cell nuclei were counterstained with DAPI (blue). Scale bar: 40 μm. Three independent biological experiments were performed (*n* = 12)

To overcome the differentiation deficiencies of FXS- brain organoids, we chose to use a guided cortical differentiation protocol implementing small molecule inhibitors of the Wnt and TGFβ signaling pathways, whose role in driving neuroectodermal fate is established [8, 37, 41–43]. Indeed, improvement was evident by increased organoid size and the presence of well-defined translucent epithelium in FXS-organoids, albeit at a lower yield (Fig. 2A). Immunofluorescence quantification analysis of the neuronal markers NeuN and TUBB3 revealed no significant difference in expression between FXS- and IC-cortical organoids at day 30 of differentiation (Fig. 2B, S2A), with TUBB3 showing a non-significant increase in IC-organoids. By day 50, both lines expressed the early neuronal marker CTIP2, the neural progenitor marker SOX2, and the intermediate progenitor marker TBR2, with no significant differences between the FXS- and IC-organoids (Fig. 2C, S2B). These results demonstrate that applying a guided protocol facilitates the generation of FXS-cortical organoids harboring a neuronal population.

Since both the Wnt and TGFβ pathways are closely related to the ECM and involved in the regulation of ECM proteins, we sought to check whether the production and remodeling of ECM components are altered in FXS-cortical organoids.

### Identification and characterization of dysregulated proteins in developing FXS-cortical organoids

**Fig. 3:**
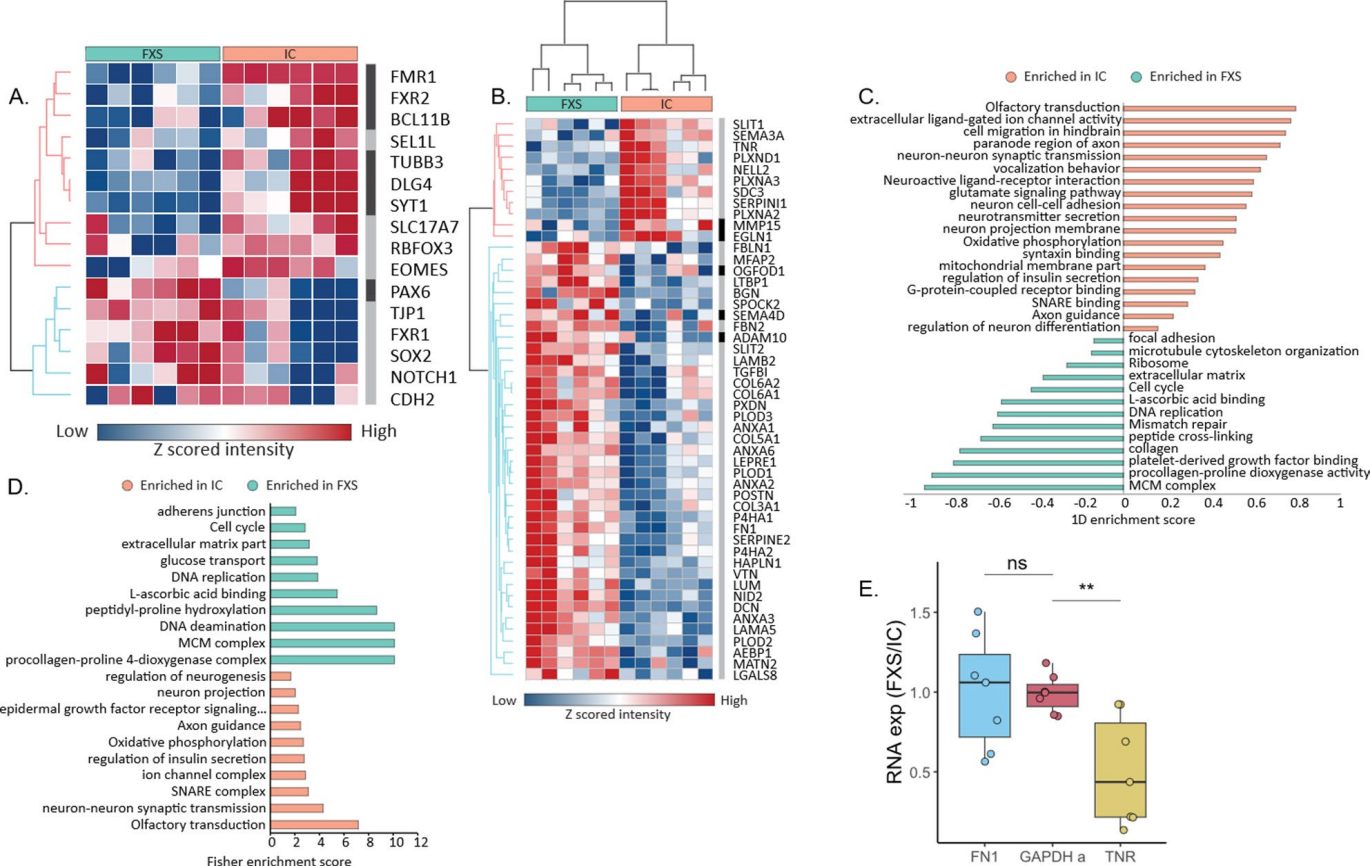
Proteomic Profiling of Cortical Brain Organoids: FXS vs. ICs. **A** Heatmap depicting Z-scored protein intensities of early brain developmental markers in IC and FXS-organoids. The levels of neuronal markers are reduced in FXS-organoids. Black row bar indicates t-test significance (Student’s t-test, FDR < 0.075). **B** Differential analysis of Matrisome proteins in FXS-organoids. Heat-map shows Z-scored protein intensities in IC and FXS-organoids. Black bars indicate proteins with fold change (FC) < 1.5. Only Matrisome proteins passing the student’s t-test (FDR < 0.075) are shown (n_FXS_=6, n_IC_=6). **C** Pathway analysis shows enrichment of cell cycle and ECM components in FXS-organoids. Selected processes from the 1D annotation enrichment test (Benjamini Hochberg FDR, q-value < 0.02) are shown. Gene annotations, including GOBP, GOMF, GOCC and KEGG pathways, were sourced from Uniport. **D** Pathway analysis highlights the enrichment of cell cycle and ECM components in FXS. Selected processes using the Fisher Exact test (Benjamini Hochberg FDR, q-value < 0.02) on significantly upregulated or downregulated proteins (Student’s t-test, FDR < 0.075) against a background of 8059 quantified proteins. **E** mRNA expression of differentially expressed ECM proteins analyzed by qRT-PCR at day 50, in FXS and control cells, IC served as a negative control for normalization. Three independent biological experiments were performed (n_FXS_=7, n_IC_=7), and values are presented as mean± Standard error. **P < 0.01; t-test

To identify dysregulated proteins in our FXS-cortical organoids, we conducted mass spectrometry-based proteomics on day 50 FXS- and IC- samples. A total of 8311 proteins were identified. Notably, there were no significant differences in the total number of proteins quantified between the two groups (Supp. Figure 1A). However, principal component analysis showed a clear separation between FXS- and IC-organoids in the first two components, indicating that *FMR1* silencing leads to global proteomics changes (Supp. Figure 1B). We first conducted a detailed comparison of early brain developmental markers between the FX and IC groups. While most markers were present in both groups, several key markers were significantly depleted in FXS-cortical organoids (Fig. 3A), including the mature neuron marker DLG4 (PSD-95), the common neuronal marker β-tubulin III (TUBB3), Synaptotagmin-1 (SYT1), an integral membrane protein of synaptic vesicles, and BCLL1B, the gene encoding the CTIP2 protein which marks intermediate progenitors. Importantly, at an earlier timepoint (day 30) TUBB3 and CTIP2 expression was not significantly different between FX and IC (Fig. 2B). Notably, the neural progenitor marker PAX6 was elevated in FXS-organoids compared to IC. We suggest these alterations stem from a delay in differentiation and neuronal maturation of FXS-organoids. This delay becomes statistically significant as normal development progresses (from day 30 to 50), a hypothesis strengthened by the higher expression of more mature neuronal markers (DLG4, SYT1)1 in IC-organoids compared to FXS-organoids on day 50.

To gain a deeper understanding of the ECM changes occurring in FXS-cortical organoids, we analyzed Matrisome proteins, including ECM and ECM-associated components [44]. We observed significant dysregulation of multiple ECM proteins in FXS-organoids compared to ICs, including semaphorin 4D (SEMA4D) and a disintegrin and metalloproteinase 10 (ADAM10) which are known to play pivotal roles in neurodevelopment [45, 46]. 

Our proteomics analysis also revealed the enrichment of various other ECM components, (Fig. 3B), including structural proteins such as collagens (COL3, COL5, COL6), fibulin 1 (FBLN1), laminin (LAM), and lumican (LUM). The ECM plays a crucial role in directing cell fate and promoting stem cell niches for self-organization, differentiation, and organoid expansion, and our data strongly suggests its involvement in organoid fate through cell–matrix interactions.

Interestingly, Fibronectin (FN1) and tenascin R (TNR) are shown here for the first time to be altered in FXS (Fig. 3B). Both proteins are known to have a critical role in neural cell migration, proliferation, adhesion and differentiation, synaptic plasticity, and more crucial neurodevelopmental processes [47, 48]. Notably, while the reduction in TNR was observed in RNA qPCR, the increase in FN1 was not, indicating that some changes occur at the protein level (Fig. 3E).

To further explore the alterations in FXS-organoids, we identified 1500 significantly differentially expressed proteins using student’s t-test. Pathway enrichment analysis using 1D (Fig. 3C) and Fischer (Fig. 3D) annotation methods showed several important pathways that were altered. Remarkably, ECM-related pathways were upregulated in both methods, indicating the substantial difference in ECM formation in FXS-organoids. Additionally, multiple pathways associated with neurogenesis and synaptic transmission were downregulated in FXS-organoids, whereas processes relating to cell cycle were upregulated, corroborating our findings of neuronal marker expression depletion and further illustrating a preference for proliferation over differentiation in FXS-organoids.

### Altered ECM deposition affects ECM-cell interactions

**Fig. 4:**
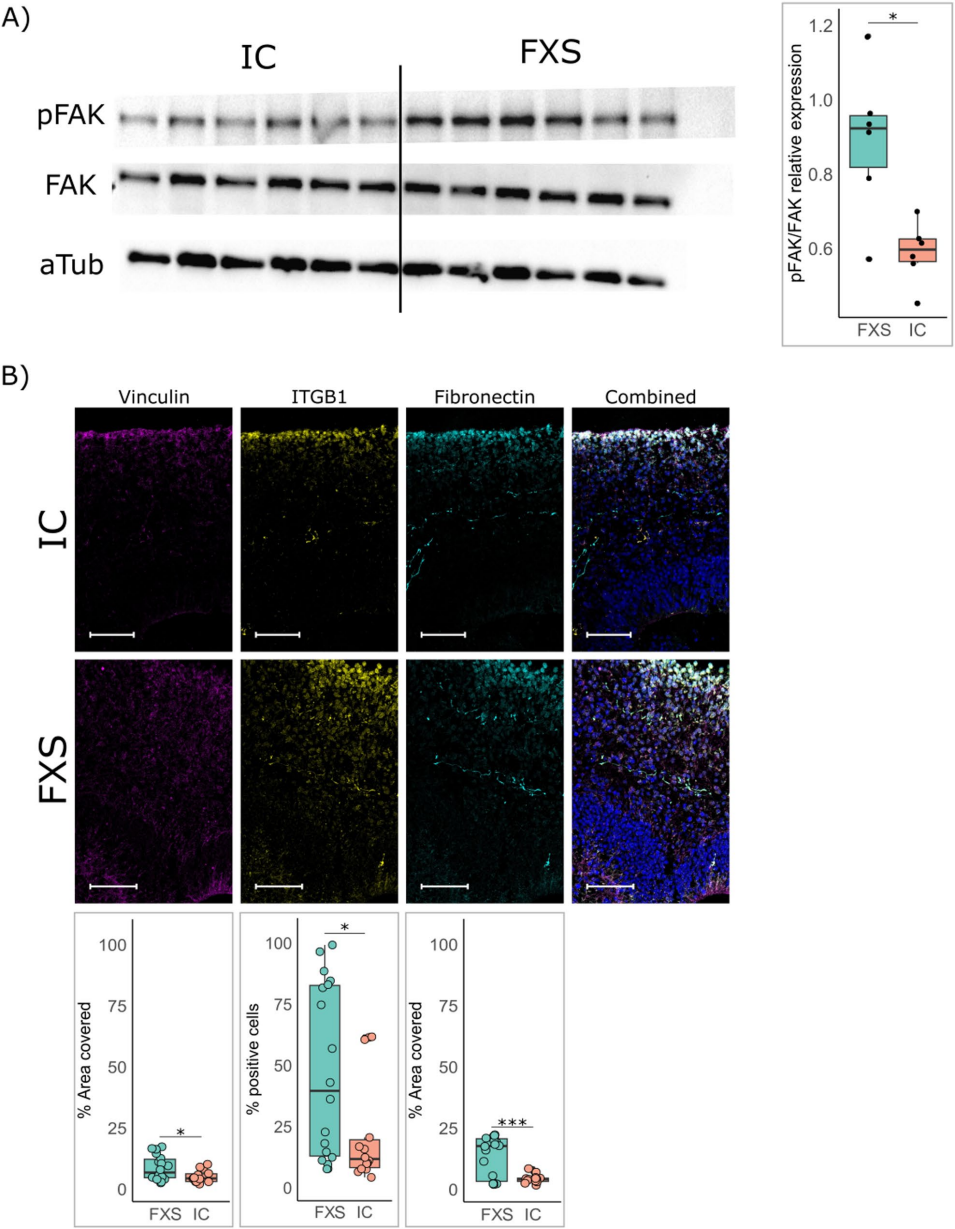
Altered ECM Deposition Affects ECM-Cell Interactions. **A**Western blot analysis of day 50 organoids for phosphorylated FAK and its quantification. *p*-value = 0.012, two-sample t-test, (*n* = 6). **B** Representative immunohistochemistry images and quantification of Vinculin (magenta), ITGB1 (yellow), and FN1(aqua) at day 50 of cortical organoid differentiation. Cell nuclei were stained with DAPI shown in blue. Area covered is the fraction of extracellular area covered by signal divided by selected area of the organoid. (nFXS=18, nIC=14) Scale bar: 60 μm.

Due to its established role in neurodevelopment, FN1 deposition was further studied using immunohistochemistry. Confocal imaging revealed significantly elevated FN1 levels in FXS-organoids compared to ICs (Fig. 4B), similar to the trend shown in proteomic analysis (Fig. 3B).

The higher levels of FN1 and other ECM proteins in FXS-brain organoids identified by the proteomics analysis (Fig. 3B) can affect a plethora of cellular processes that can be activated by cell-ECM interactions. The changes observed in the cell adhesion and focal adhesion pathways (Fig. 3C, D) prompted us to test the prominent pathways that are triggered by the ECM components. Fittingly, we observed a significant increase in β1 integrin (ITGB1) (Fig. 4B), as this subunit of integrins is indeed involved in many ECM mediated signaling interactions. We further investigated the impact of ECM alterations on the activation of focal adhesion kinase (FAK), a key signaling molecule involved in regulating cell growth, survival, proliferation, migration and invasion [49]. Western blot analysis of FAK and its phosphorylated state revealed increased activation of FAK signaling in FXS-organoids, evident by pFAK/FAK ratio (Fig. 4A). pFAK, along with vinculin, plays a crucial role in the complex regulation of focal adhesions, which are essential for cell adhesion, migration, and survival. The enhanced activation of FAK downstream signaling in FXS-organoids was further confirmed by significantly higher vinculin levels in FXS- vs. IC-organoids as seen by immunofluorescence quantification (Fig. 4B, S2C). Remarkably, FN1, ITGB1 and vinculin displayed differential distribution in FXS-organoids. These proteins are abundant throughout the entire FXS organoid (Fig. 4A), whereas they were mainly localized to the marginal layer in the IC samples. These cellular processes, along with altered kinases activity, have been previously demonstrated to contribute to FXS pathology [50]. Notably, our findings now demonstrate the upstream impact of ECM molecules on these processes.

## Discussion

FXS is the most common cause of inherited intellectual disability and currently has no cure. This challenging genetic disorder remains elusive due to scarcity of models that recapitulate its developmental aspect, including the gradual silencing of *FMR1*, the phenotypic variability among patients, and technical limitations in developing models that faithfully mimic human neurological conditions.

In this work we established and characterized an advanced cortical organoid model for FXS, providing a system that better resembles human brain development. For this purpose we utilized our unique platform of isogenic human embryonic stem cell lines derived from preimplantation genetic diagnosis (PGD) embryoes. We successfully generated FXS-organoids that developed up to 50 days by supplementing the early differentiation medium with Wnt and TGFβ small- molecule inhibitors. These pathways have been previously implicated in hippocampal development in fetuses [42, 43], generation of functional hippocampal tissues in vitro [37], and the improvement of FXS-neuron migration [41].

Previous studies have utilized iPSCs generated from patients or FMRP-knockout hiPSC lines [51, 52] to generate brain organoids as a model of FXS. These studies have shown that absence of FMRP leads to abnormal proliferation, cell cycle alterations, increased gliogenesis, and an imbalance of neural and glial development.

As shown here, substantial differences exist between IC- and FXS-organoids. Our analyses revealed significant alterations in protein levels of neural progenitor and neuronal markers between FXS- and IC- organoids at various stages of differentiation, as observed through both immunofluorescence and proteomics analyses. Specifically, we found that neuronal markers such as DLG4, TUBB3, SYT1 and CTIP2 levels were reduced in FXS-organoids on day 50. These findings suggest delayed and reduced fate commitment in early stages of neurodevelopment in FXS-organoids. This phenotype has been corroborated by other studies in FXS models [34, 53, 54]. In fact, we postulate that this penchant of FXS-cell for proliferation over differentiation is the reason we struggled to produce FXS-organoids using Lancaster’s cerebral protocol, which is less guided and contains fewer morphogens. Collectively, these studies indicate altered neurogenesis in the developing FXS brain, warranting further investigation.

Cell-cell communication and migration during early brain development are fundamental prerequisites for the expansion of the human cerebral cortex. ECM plays a vital role not only in providing structural support to cells but also in relaying signals that promote these important processes. Our mass spectrometery analysis showed altered expression of several ECM molecules and their associated pathways in FXS-organoids.

Among the many altered ECM molecules identified in FXS-organoids, we focused on fibronectin (FN1), which was upregulated in FXS-organoids, and its co-signaling molecules. Our findings clearly show the concurrent elevation of ITGB1, one of the primary cell membrane receptors of FN1. It is already known that the ECM-integrin axis can activate focal adhesion plaques and cytoskeletal changes [55]. We also uncovered elevated levels of vinculin, thus pointing to altered adhesion/migration dynamics.

Finally, FAK is a central molecule in many cellular signaling pathways, including cell survival and cell cycle regulation. Phosphyrlation of FAK at Try397 is an important step in promoting migration and proliferation in several systems [49]. Our data showed an elevated pFAK/FAK ratio in FXS-organoids, with cell cycle being a prominent pathway disrupted in FXS-organoids. Collectively, these results show an interesting concurrent increase in FAK phosphorylation, FN levels, and integrin-mediated adhesion pathway activity in FXS. Focal adhesion proteins and cell adhesion pathways influence a myriad of processes including cell-cell communication, migration and proliferation, and the mechanism by which these changes contribute to FXS pathology requires further study.

To support organoids expansion and growth in later stages organoid media was supplemented with 1% Matrigel. Dissolved Matrigel in media does not polymerize and only coats the surface of organoids [56], and is therefore unlikely to have influenced our analyses, which showed differential ECM distribution within organoids and total quantification and differential expression of ECM proteins and pathways which are not among the major components in Matrigel.

Based on their origin from human embryos, our hESC-derived 3D organoids capture human brain development with greater fidelity than iPSC-based systems. Specifically, our model successfully recapitulates the developmentally-regulated silencing of the FMR1 gene, which is absent in iPSC models. Furthermore, these cortical organoids offer a more faithful model of FXS compared to traditional 2D cell cultures or knockout mouse models. As complex 3D multicellular systems, they better recapitulate critical features of the developing human brain, including cellular diversity, tissue architecture, cell-ECM interactions, and developmental dynamics. The identical genetic background of our IC- and FXS-clones allows for direct comparison between them, significantly enhancing the reliability and reproducibility of experimental results. Using isogenic clones enabled us to elucidate of how the FMR1 gene mutation drives early neurogenesis and affects subsequent disease development. Nevertheless, due to the inherent impact of genetic background on processes such as organoid development, the reliance on data derived from a single isogenic pair of hESC lines limits the generalizability of our findings. Therefore, the observed differences should be interpreted cautiously, as some aspects may be clone-specific.

Overall, our organoid model presents a valuable platform for advancing the understanding of FXS pathology. FXS- and IC-organoids may also serve as a robust platform for high-throughput drug screening, facilitating the identification of compounds that may alleviate FXS symptoms and accelerate the development of potential therapies in a controlled, human-relevant system.

## Methods

### Human embryonic stem cell culture and isogenic cell lines

The use of spare in vitro fertilization (IVF) derived embryos following PGD for the generation of hESCs was approved by the Israeli National Ethics Committee (7/04–043) and in accordance with the guidelines released by the Bioethics Advisory Committee of the Israel Academy of Sciences and Humanities. All experiments were conducted using a pair of FXS and its IC sub-clones that were derived from the original Lis_FXS6 hESC line from relatively early passage (p 40–55) that present CGG repeats from the normal to the full mutation range. Clone 8 A has > 200 CGG repeats and thus served as the full mutation clone and clone 7B has < 50 CGG repeats and thus served as its IC that share the same genetic background. Full characterization of these isogenic clones is described in Gildin et al. [38], including CGG repeats number analysis by a specific designed PCR CGG repeat number assay and by the AmplideX PCR/CE *FMR1* Reagents (Asuragen), confirmation of their polymorphic markers by CA repeats analysis known to identify their parental Lis_FXS6 line, and the expression of pluripotent markers.

hESC were cultured in mTeSR1 medium (Cat No. 85850, STEMCELL Technologies, Vancouver, Canada) supplemented with 100 µg/mL Primocin (Cat No. ant-pm-1, InvivoGen, CA, USA) on Geltrex (Cat No. A1413202, Thermo Fisher Scientific, MA, USA) coated plates, at 37 °C in a 5% CO_2_ atmosphere and media was changed every other day. hESC were passaged using Accutase (Cat No. SCR005, Merck, Germany) and seeded with 5 µM ROCK inhibitor Y-27,632 (Cat No. 1683, Axon Medchem, VA, USA) for one day to increase survival. Cells were frozen using CryoStem hPSC Freezing Medium (Cat No. 05-710-1E, Biological Industries, CT, USA) using a CoolCell LX (Cat No. BCS-405, Biocision, CA, USA) overnight at -80 °C, and then in vapor-phase liquid nitrogen for long-term storage.

### Generation of brain organoids

For the unguided protocol, brain organoids were generated by the un-guided protocol as described [39, 40], with one exception: EBs were generated by plating 8.1 × 10^5^ single cells suspension on a MicroTissues^®^ 3D Petri Dish^®^ micro-molds size L, 9 × 9 array, that fits 12 well plates (Sigma-Aldrich). 100 µL of cells suspension was delivered into MicroTissues 3D Petri dishes, which resulted in a final concentration of 10,000 cells per micro-well/EB. Plates were placed at 37 °C in a 5% CO_2_ atmosphere for 15–20 min to enable sinking of cells, then the 12-wells were carefully flooded with 2 ml media for further growth and totally replaced according to the protocol. For morphological analysis of the organoids, Frames were captured using ×4 magnification. Organoid’s diameter (mm) was measured manually on bright-field frame with CellSence measuring software. In each time point (days 6, 10, 15 and 21) > 20 organoids were measured from a minimum of 13 visual fields. Data from four independent biological replicates from each cell line were collected. For the guided (cortical) approach, cortical organoids were generated as described [41]. Briefly, cells were dissociated using Accutase when they reached 70–80% confluent. The colonies were pelleted by centrifugation and resuspended in fresh mTESR1. The colonies were mixed properly to make single cells. The cells were then plated onto a 96 well V bottom plates (Sumilon PrimeSurface plates; Sumitomo Bakelite) at a density of 10,000 cells/well in S1 medium (DMEM, 20% v/v knockout serum replacement, 0.1mM NEAA, 1mM Sodium pyruvate (Thermo Fisher Scientific), 0.1 mM β-mercaptoethanol, 1% Penstrep) in the presence of small chemical inhibitors of the TGFβ and Wnt pathways; 5µM SB431S42 and 3µM IWR-1 (Calbiochem), and 80 µM rock inhibitor. The cells were allowed to form aggregates of embryonic bodies (EBs) in a humidified 5% CO_2_, 95% air incubator at 37 °C. Medium was changed every other day until day 10 with a final concentration of 20µM rock inhibitor followed by S1 medium without rock inhibitor for another 8 days. From day 18–22 the EBs were supplemented every alternate day with S2 medium (DMEM/F-12, Chemically Defined Lipid Concentrate (CDLC; Invitrogen), N2 supplement (Invitrogen, cat. no. 17502048), 1x Glutamax, 1% Penstrep). The EBs were then transferred to a low adhesion culture dish in S2 medium supplemented with B27 without Vitamin A (Invitrogen, cat. no. 12587010), 1% Matrigel(Corning, cat. no. 354230). The organoids were grown from then on in suspension culture with constant mixing using an orbital shaker fitted inside a 37 °C CO2 incubator. The medium was replaced every third day. The media was supplemented with 1% B27 (Invitrogen, cat. no. 17504044) after day 60. The organoids were observed under microscope regularly for proper growth.

### Organoids measurements

Frames of brain organoids were captured using ×4 magnification. Organoid’s diameter (mm) was measured manually on bright-field frame with CellSence measuring software. In each time point (days 6, 10, 15 and 21) > 20 organoids were measured from a minimum of 13 visual fields. Data from four independent biological replicates from each cell line were collected. Graphs and error bars represent as mean± Standard error and are considered statistically significant when *P* < 0.05 by unpaired two-tailed Student’s t-test using PRISM software.

### Immunofluorescence and quantification of expression

Organoids were washed briefly once with cold PBS and fixed immediately in 4% PFA for 2 h and then overnight (ON) in 2% PFA at 4 °C. For bigger tissues, the ON fixation was performed in 4% PFA. The tissues were then washed thrice for 15 min with PBS and transferred to a 30% sucrose solution. The tissues after sunken were placed on a mold and filled with optimal cutting temperature (OCT) and snap froze using a mixture of dry ice and ethanol. The blocks were stored in -80 °C till sections of 20 µM were made in a cryostat. The sections were washed to remove the OCT, blocked with 20% normal donkey serum (NDS) with 0.2% Triton X 100 for 90 min. Freshly prepared primary antibodies in 2% NDS were added to the sections and incubated ON in a humidified chamber at 4 °C. The sections were then washed thrice with PBS and incubated with suitable secondary antibodies for an hour. The sections were again washed and mounted using immunomount. The sections were imaged using Dragonfly spinning disk confocal microscope and processed using Thermofisher Amira software (2023.1).

Cortical regions of each organoid were manually selected and analyzed. Separate analysis procedures were used for nuclear\cellular markers (CTIP2, Sox2, TBR2, NeuN, ITGB1) and for extracellular markers (Vinculin, Tuj1, Fibronectin). Nuclear analysis involved detection and separation of the nuclei using the DAPI channel, then creating a mask for individual nuclei. This mask was used to measure the median intensity of all other channels over the generated mask. Cells were determined positive if their median intensity was over a set threshold above the background. The proportion of positive cells was thus calculated for each nuclear channel.

Extracellular analysis involved measuring the proportion of the selected area covered by the extracellular signal. A constant threshold was applied thus creating a single mask per region of interest. The nuclei area was removed to isolate the signal originating from the ECM (Shown in S4). Image analysis pipelines and R scripts for statistical analysis can be found in the supplementary information.

### Western blot analysis

Selected organoids with proper morphological features at various time points were collected, quickly washed three times with PBS, and immediately processed for protein isolation. Organoids were homogenized in 5% SDS in TRIS-HCl (pH 7.5) using a bead homogenizer with soft tissue homogenizing tubes. The homogenate was divided for use in western blotting and mass spectrometry and stored at -80 °C until further analysis. Protein samples, mixed with sample buffer, were separated by 10% SDS-polyacrylamide gel electrophoresis (SDS-PAGE) and transferred to nitrocellulose membranes (0.2 μm; Thermo Fisher Scientific). The membranes were blocked with 5% BSA and incubated overnight at 4 °C with primary antibodies. After three washes with TBST, the blots were incubated at room temperature for 1 h with secondary antibodies. Following three additional TBST washes, the blots were developed using chemiluminescent Pico substrate (Cat No. 34577, Thermo Fisher Scientific). The developed blots were imaged using a chemiluminescent gel documentation system (ChemiDoc XRS+ System, Bio-Rad).

### Liquid chromatography with tandem mass spectrometry (LC-MS/MS) and pathway analysis

A total of 100 µg of protein from each sample was processed using an S-trap (Protifi). The resulting peptides were fractionated offline using high-pH reversed-phase liquid chromatography, followed by online nanoflow liquid chromatography (nanoAcquity) coupled to high resolution, high mass accuracy mass spectrometry (Exploris 480). Each sample was analyzed on the instrument separately in a random order in discovery mode.

Raw data was processed with MaxQuant (v1.6.6.0) [57]. The data was searched with the Andromeda search engine [58]against the human proteome database appended with common lab protein contaminants and the following modifications: Carbamidomethylation of cysteine as a fixed modification and oxidation of methionine and acetylation of protein N-termini as variable ones. The LFQ (Label-Free Quantification) intensities were calculated and used for further calculations using Perseus (v1.6.2.3) [59]. Decoy hits, proteins that were identified based on a modified peptide, and potential contaminants were removed. Data was log2 transformed and only proteins with quantification in 50% of the saples in at least one group were retained. Missing values were imputed based on normal distribution with a width of 0.3 and downshift of 1.8. Differentially expressed proteins between control and FXS samples were extracted by performing Student’s t-test (Permutation based FDR cutoff of 0.075). For pathway enrichment analysis, gene annotations including GOBP, GOMF, GOCC, and KEGG pathway were added from Uniport. ECM proteins were annotated according to categories defined by Naba et al. [44]. Fisher Exact test was performed on the significantly changing proteins with an FDR cutoff of 2% with 8059 proteins as background. 1D annotation enrichment test was performed on the fold change values between control and FXS samples (Benjamini Hochberg FDR, q-value < 0.02).

### Statistical analysis

For all experiments, three independent experiments were carried out unless otherwise stated. All statistical analysis was conducted in Prism software (La Jolla, CA, USA). Statistical significance was determined by paired or unpaired two-tailed Student’s t-test or one-way ANOVA. Differences were considered statistically significant when *p* < 0.05.

## Supplementary Information

Below is the link to the electronic supplementary material.

Supplementary Material 1

## Data Availability

Data and supporting materials are not openly available due to their large size and are available from the corresponding author upon reasonable request. Data is located in controlled access data storage at the Weizmann Institute of Science.

## Abbreviations

ASD: Autism spectrum disorder
CDLC: Chemically defined lipid concentrate
COL: Collagens
ECM: Extracellular matrix
FAK: Focal adhesion kinase
FBLN1: Fibulin 1
FC: Fold change
FMRP: Fragile X messenger ribonucleoprotein
FN1: Fibronectin
FX: Fragile X
hESC: Human embryonic stem cell
hPSC: Human pluripotent stem cells
iPSC: Induced pluripotent stem cell
ITGB1: integrin β1
IVF: In vitro fertilization
LAM: Laminin
LFQ: Label–free quantification
LUM: Lumican
NDS: Normal donkey serum
NPC: Neural precursor cells
OCT: Optimal cutting temperature
ON: Overnight
PGD: Preimplantation genetic testing
SDS: PAGE–SDS polyacrylamide gel electrophoresis
TNR: Tenascin R
