# Supplementary material for: Altered ECM deposition and cell adhesion signaling in a human cortical organoid model of fragile X syndrome
Source: Mol Brain. 2026 Apr 15;19:49. doi: 10.1186/s13041-026-01280-8 (PMC13308187; doi:10.1186/s13041-026-01280-8)

**Supplementary Materials**

Supplementary Figure 1:


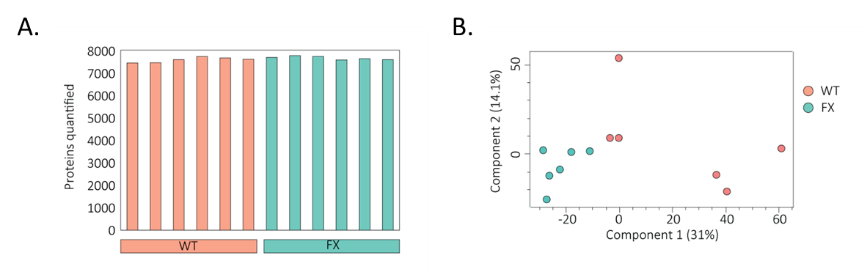


Supplementary figure 2: whole frame images


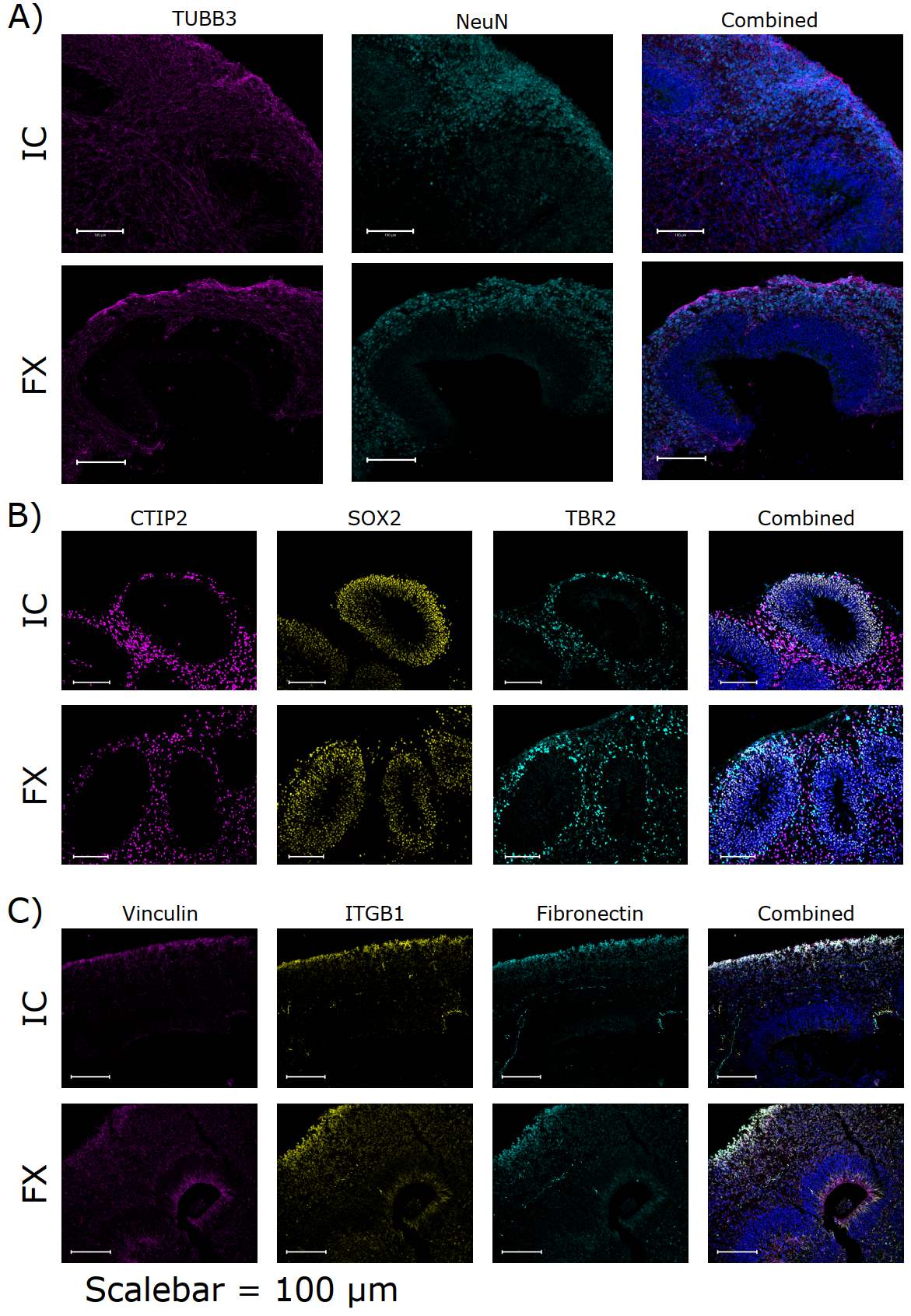


Supplementary figure 3: threshold analysis pipeline for ECM proteins:


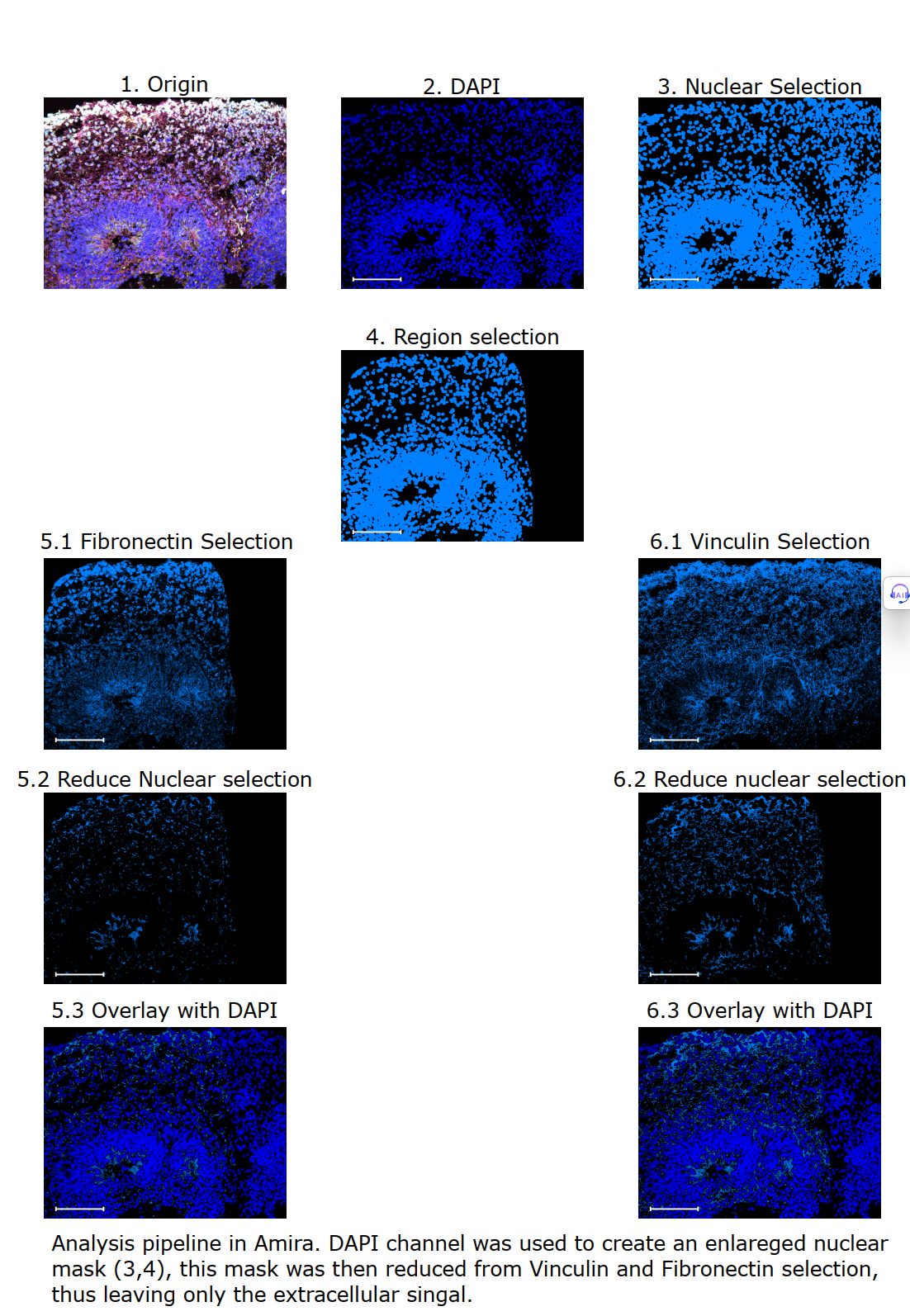

Supplement: Supplementary file 1 — Supplementary Material 1 [file 13041_2026_1280_MOESM1_ESM.docx]
